# Ottawa Medico-Chirurgical Society

**Published:** 1849

**Authors:** 


### ARTICLE VI.

### OTTOWA MEDICO CHIRURGICAL SOCIETY.

This Association, we learn by a letter from the Secretary, was organized on the 1st of January last, by the election of Allen H. Howland, M. D., President; M. A. Gooding, M. D., Vice President; E. A. Guilbert, M. D., Secretary; and J. Pearson, M. D., H. Rosenkrans, M. D., and Joseph Stout, M. D., Censors. It convenes every two weeks, and its meetings have so far been interesting. The code of ethics of the "American Medical Association" was adopted and each member pledged to live up to its precepts. We wish our friends success in their efforts.

